# Comparative In Vivo Study of Solid-Type Pure Hyaluronic Acid in Thread Form: Safety and Efficacy Compared to Hyaluronic Acid Filler and Polydioxanone Threads

**DOI:** 10.1007/s00266-023-03614-6

**Published:** 2023-08-29

**Authors:** Jong-Ho Kim, Man Wong Han, Myoung-Han Lee, Dong-Keon Kweon, Young Jin Park, Chan Yeong Heo

**Affiliations:** 1 Department of Plastic and Reconstructive Surgery, Seoul National University College of Medicine, Seoul National University Bundang Hospital, 82 Gumi-ro 173beon-gil, Bundang-gu, Seongnam, 463-707 Korea; 2 Jinwoo Bio Co. Ltd., Yongin si, Korea; 3 Ksamsung Plastic Surgery, Seoul, Korea

**Keywords:** Solid-type HA thread, Hyaluronic acid filler, Polydioxanone thread, in-vivo study

## Abstract

**Introduction:**

Although various products are commonly used for skin rejuvenation, solid-type hyaluronic acid (HA) as an injectable form has not been researched or utilized. This study aimed to demonstrate the safety and efficacy of solid-type HA in thread form, which differs from the conventional gel-type HA commonly used.

**Method:**

Solid-type HA threads, conventional HA fillers, and polydioxanone (PDO) threads were inserted into the dorsal subcutaneous layer of mice. Photographs were taken on days 0, 1, 3, and 7, and on day 7, the samples were harvested for histological analysis. Inflammatory reactions and detection of collagen were confirmed through tissue staining, and real-time PCR was conducted to quantify collagen synthesis.

**Results:**

In the histological analysis, the PDO threads exhibited a greater inflammatory response compared to the HA threads. Masson’s trichrome staining revealed a higher degree of collagen synthesis in the HA thread group compared to the HA filler group. While collagen type 1 expression was significantly higher in the PDO thread group than in the HA thread group, the HA thread group showed higher expression levels of collagen type 3. Furthermore, the PDO thread group demonstrated a statistically significant increase in TGF-β1 compared to the HA group.

**Conclusion:**

This in vivo study demonstrated the stable application of solid-type pure HA threads and their potential for inducing collagen production, while also yielding a low inflammatory response. The findings highlight the promising applications of solid-type HA in the field of cosmetic dermatology.

**No Level Assigned:**

This journal requires that authors assign a level of evidence to each submission to which Evidence-Based Medicine rankings are applicable. This excludes Review Articles, Book Reviews, and manuscripts that concern Basic Science, Animal Studies, Cadaver Studies, and Experimental Studies. For a full description of these Evidence-Based Medicine ratings, please refer to the Table of Contents or the online Instructions to Authors www.springer.com/00266.

## Introduction

Hyaluronic acid (HA) is a natural substance found in the human body, particularly in connective tissues, skin, and eyes [1]. It is a glycosaminoglycan, a type of molecule composed of both sugar and amino acid building blocks. HA has several important functions in the body, including providing lubrication and cushioning for joints, maintaining skin elasticity, and helping with wound healing. It is also a key component of the extracellular matrix, which provides structural support to cells and tissues [2]. HA has gained significant attention in the field of cosmetic dermatology due to its remarkable ability to improve skin hydration and moisture retention. When applied topically, it can help reduce the appearance of fine lines and wrinkles, as well as improve skin texture and tone [3, 4]. In injectable form, it can be used as a filler to add volume and contour to the face [5, 6].

Although various products such as HA fillers, polydioxanone (PDO) threads, and polylactic acid (PLA) threads are commonly used for skin rejuvenation, solid-type HA as an injectable form has not been extensively researched or utilized [7–9]. Currently, there have been advancements in the development of hyaluronate fibers, which allow for the production of solid-type HA that can be inserted [10, 11]. Therefore, the aim of this study was to demonstrate the safety and efficacy of solid-type HA in thread form, which differs from the conventional gel-type HA commonly used.

## Methods

### Threads and Fillers

**Fig. 1:**
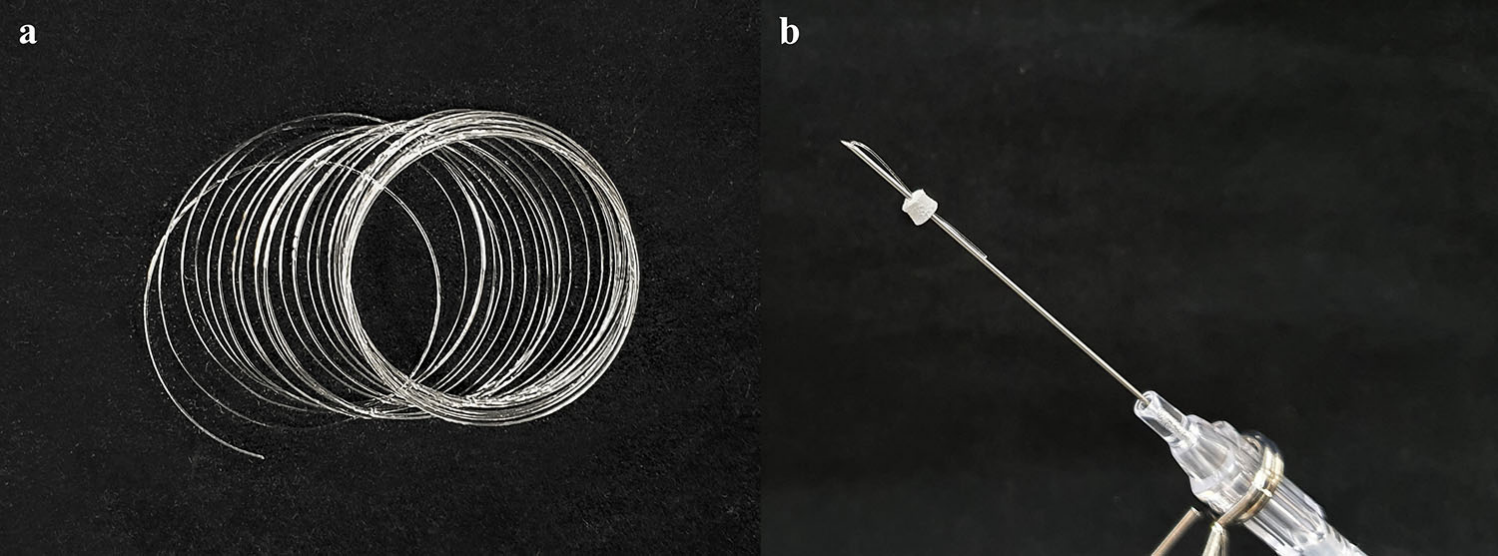
Solid-type hyaluronic acid threads (**a**). Needle for hyaluronic acid thread insertion (**b**)

The study utilized an absorbable PDO thread (SkinMedience, Anyang, Korea) and a non-crosslinked HA filler (Yvoire-Hydro; LG Life Sciences, Seoul, Korea) as the control group. The experimental group consisted of a newly manufactured non-crosslinked hyaluronic acid solidified HA thread (Jinwoo Bio, Yongin, Korea) (Fig. 1) [10, 11]. HA thread and absorbable PDO thread were inserted as 2-0 USP (thickness: 0.34 mm) on 23G needle.

### Animal Preparation and Study Procedures

Animals were raised in a separate facility under controlled environmental conditions, including a temperature of 24 °C, relative humidity of 55%, and a 12-hour light/dark cycle, with a minimum acclimation period of 1 week prior to the start of the experiment.

The study consisted of two separate experiments. In the first experiment, a comparative study was conducted to evaluate the effectiveness and safety of HA threads versus HA fillers. The second experiment involved comparing HA threads with PDO threads to assess their efficacy in a similar context. In the first experiment, mice were chosen for accurately evaluating volume changes due to their smaller body size and thinner skin compared to rats. In the second experiment, rats were utilized due to their more discernible subcutaneous fat tissue layer, which allowed for a comprehensive histological examination of the differences between PDO and HA threads.

**Fig. 2:**
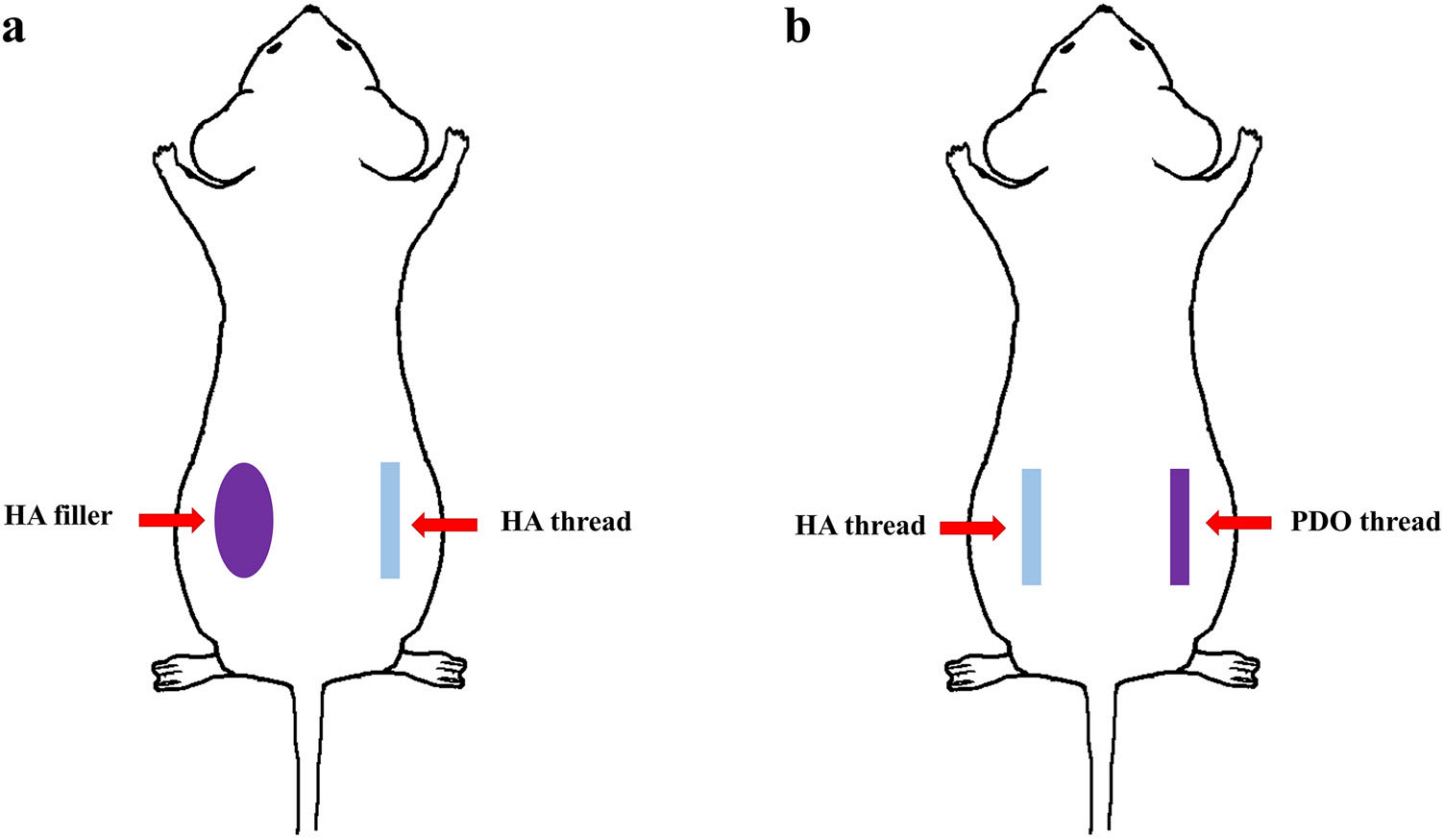
Inserted into subcutaneous layer. HA filler on the left side and HA thread on the right side (**a**). Inserted into subcutaneous layer. Two HA threads on the left side and two PDO threads on the right side (**b**)

In the first experiment, ten 7-week-old female SKH-1 hairless mice (Orient Bio, Seongnam, Korea) were purchased. After anesthetization with isoflurane, the mice dorsal skin was disinfected with povidone–iodine. HA thread (3-cm length, 0.34-mm diameter, HA 2.59 mg) and HA filler (100 ul, HA 2 mg) were inserted into the left and right lateral sides from the midline, respectively (Fig. 2). Photographs were captured on days 0, 1, 3, and 7, and on day 7, the samples were harvested for histological analysis, including the examination of the surrounding subcutaneous tissue.

In the second experiment, twenty-four 7-week-old male Sprague Dawley (SD) rats were purchased (Orient Bio, Seongnam, Korea). After undergoing the same anesthesia and hair removal process, the implantation point was marked. Using 21G, make a path at the thread insertion site in the subcutaneous layer. Based on the midline, two HA threads were inserted on the left side, and two PDO threads were inserted on the right side (Fig. 2). All threads were 20 mm in length and 0.34 mm in diameter. Half were harvested on day 7 and the other on day 14 for histological analysis, including surrounding subcutaneous tissue. The tissue sample was divided into four groups, and each part underwent staining and real-time PCR analysis.

### Histologic Analysis

Tissue specimens were fixed in 10% formalin, paraffin-embedded, and sectioned at 5 µm. Hematoxylin and eosin staining was used for histological evaluation. Additionally, the analysis included assessment of the longevity of the inserted thread or filler, evaluation of the degree of inflammatory reaction, and detection of collagen using Masson’s trichrome staining.

### Real-Time PCR

The total RNA was extracted from the tissue samples using TRI reagent (Thermo Fisher Scientific), and cDNA was synthesized using the Transcriptor First Strand cDNA Synthesis Kit (Roche) with random hexamer. The resulting cDNA samples were analyzed using SYBR Green on the Roche Cycler 480 II. The PCR primers used for amplifying COL1α1, COL3α1, and TGF-β1 were as follows: COL1α1—F: 5′-aatggtgctcctggtattgc-3′, R: 5′-ggttcaccactgttgccttt-3′; COL3α1—F: 5′-gggatccaatgagggagaat-3′, R: 5′-ggccttgcgtgtttgatatt-3′; and TGF-β1—F: 5′-tgagtggctgtcttttgacg-3′, R: 5′-tgggactgatcccattgatt-3′. The amplification conditions included an initial denaturation at 95°C for 5 minutes, followed by 45 cycles of denaturation at 95 °C for 10 seconds, annealing at 60 °C for 10 seconds, and extension at 72 °C for 10 seconds. Finally, melting curve analysis was performed from 50 to 105 °C with readings taken every 0.5 °C. Statistical comparison of the expression levels of COL1α1, COL3α1, and TGF-β1 was analyzed using paired *t*-test.

## Results

### Gross Observation

**Fig. 3:**
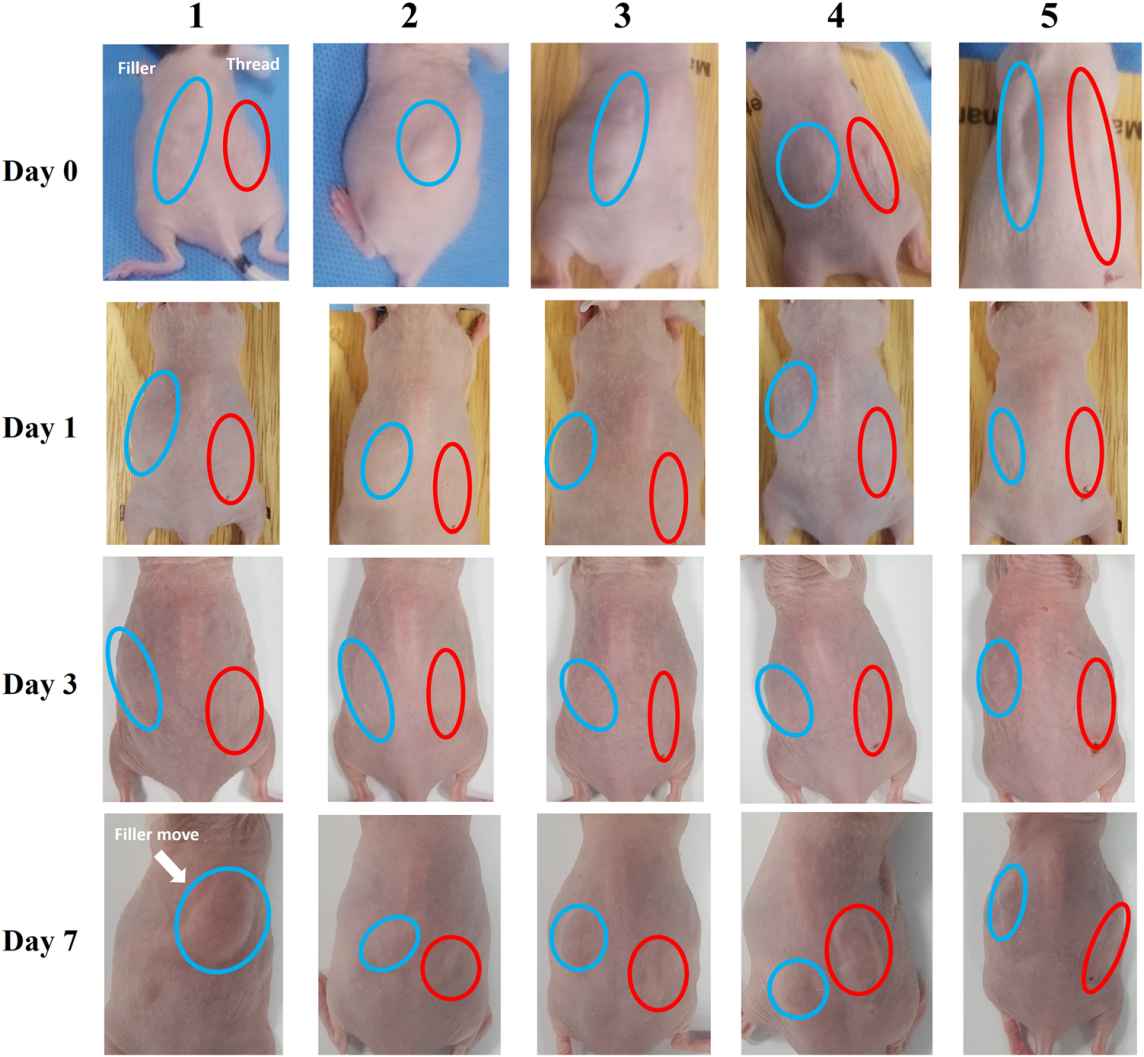
Gross changes for 7 days after HA thread and HA filler insertion. Blue circle (HA filler) and red circle (HA thread). The filler was observed visually during the 7-day period, and there was a filler shift (arrow) in some of the mice. While the threads were visible to the naked eye on the day 1, they became harder to observe on the day 2, but became visible again in some mice from the day 3 onward

Observations were made over a period of 7 days to visually track the changes in mice following insertion of the thread and filler. There were no apparent signs of inflammation observed in the HA inserted area throughout the experimental period. On day 0, a significant volumizing effect of the filler was observed, and the insertion site was visible for both filler and thread. On day 2, while the filler was still visible to the naked eye, it had decreased in size compared to day 0. The thread was difficult to observe with the naked eye. On day 3, the filler was still visible, but only a few individuals showed visible threads. On day 7, the filler and some of the thread were observed to be visible to the naked eye. In some cases, the filler had undergone a shift from its original position. The filler was more distinctly visible over time than the HA thread. The visibility of the thread starting from day 3 onward is attributed to the absorption of moisture, resulting in the HA thread forming a gel-like consistency (Fig. 3).

### Histologic Change

**Fig. 4:**
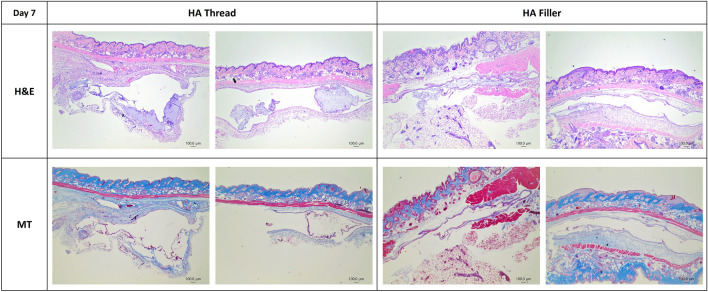
Histologic finding of HA thread and HA filler, 7 days after insertion. In H and E staining, both HA thread and HA filler showed similar levels of inflammation. In MT staining, it seems that collagen synthesis is more prominent in the HA thread compared to the HA filler

The first experiment comparing HA thread and HA filler showed that on day 7, both the HA thread and filler were not stained. This was likely due to them being washed out during the fixation process. However, the space where the hyaluronic acid was located was indirectly confirmed. In Fig. 4, it can be observed that the level of inflammatory cell infiltration is comparable between the HA thread and HA filler groups. However, the results from Masson’s trichrome staining suggest that HA threads exhibit a more pronounced enhancement of collagen synthesis compared to the HA filler group.

**Fig. 5:**
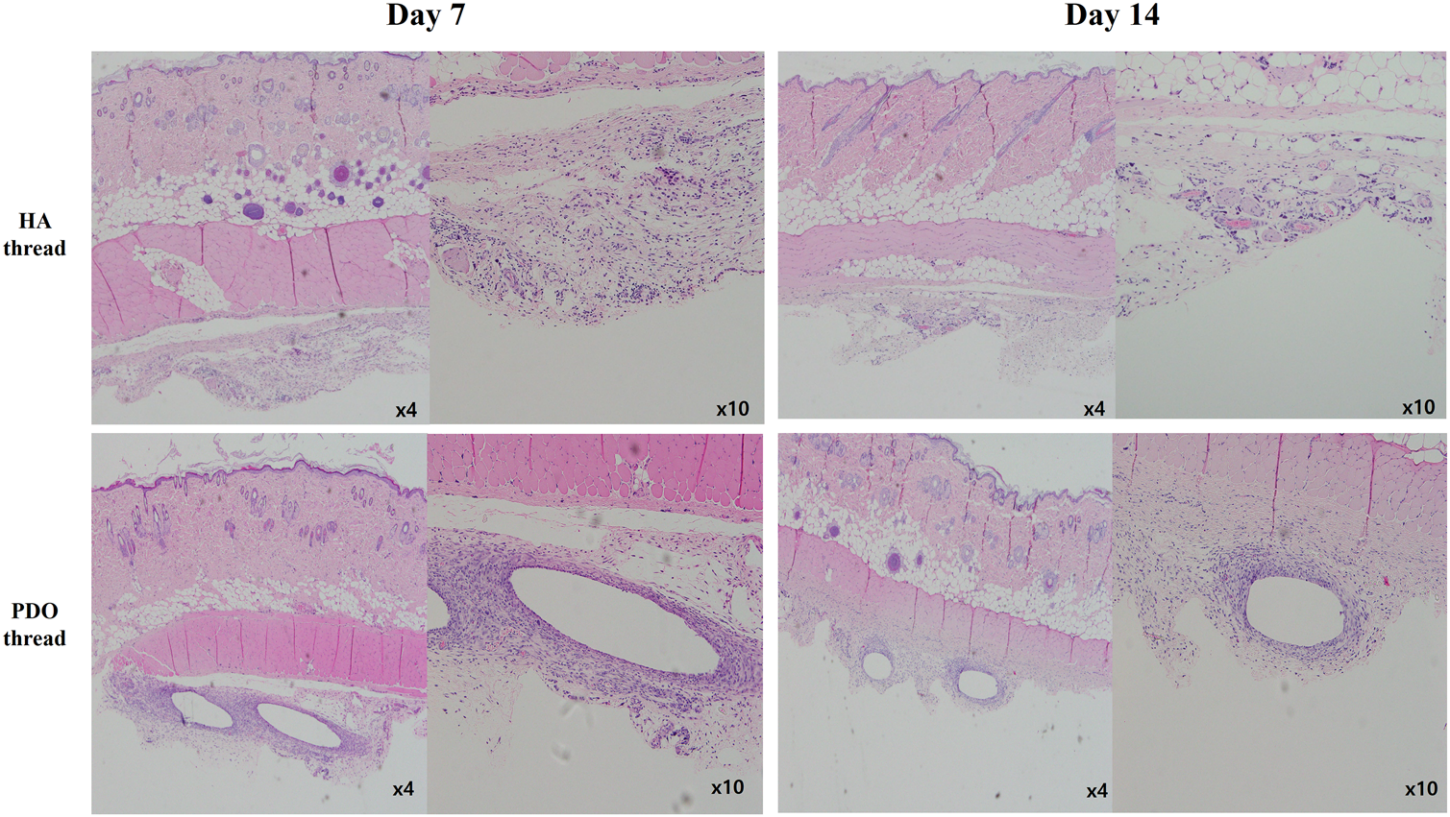
Histologic finding of HA thread and PDO thread, 7 days and 14 days after insertion. In both HA thread and PDO thread groups, the degree of inflammatory response was reduced on day 14 compared to day 7. On the same day, PDO threads show a more severe inflammatory response than HA threads

In the second experiment comparing HA threads and PDO threads, PDO threads were observed on both day 7 and day 14. Inflammatory cells and fibroblasts were observed to gather around the threads, forming a fibrous capsule-like structure. The inflammatory response was more pronounced on day 7 and decreased on day 14 in both groups. Compared to the HA threads, the PDO threads showed more inflammatory response on both day 7 and day 14 (Fig. 5).

### Collagen and TGF-Beta Analysis

**Fig. 6:**
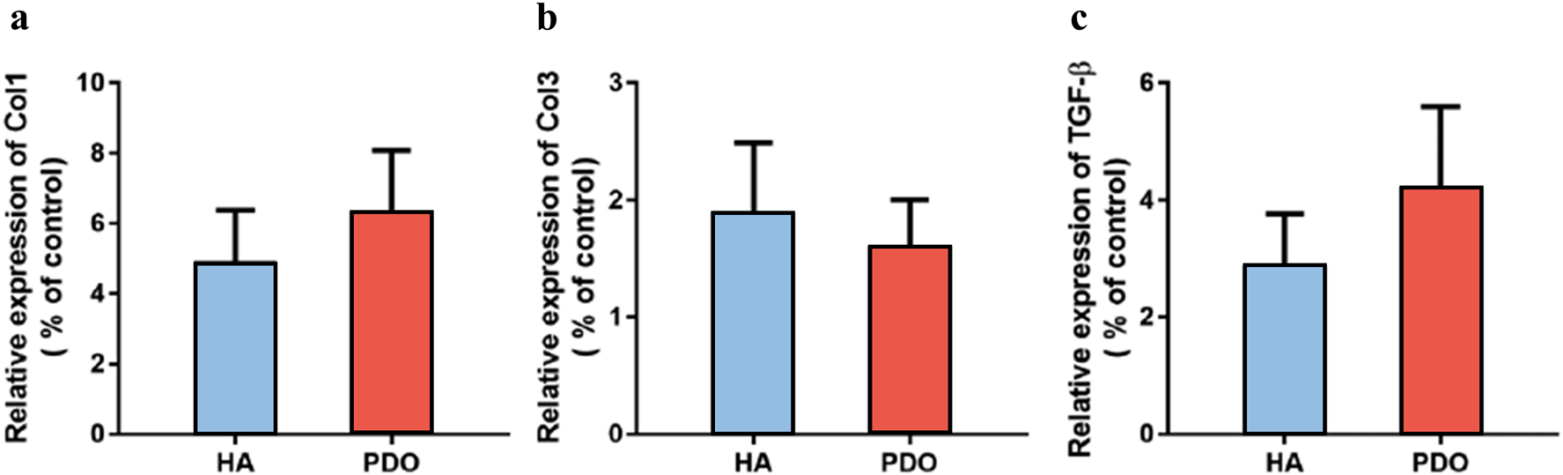
The amount of collagen type 1 (**a**), type 3 (**b**), and TGF-β (**c**) synthesis according to the type of thread measured at 7 days

The expression levels of COL1α1, COL3α1, and TGF-β1 were measured using RT-PCR. COL1α1 and COL3α1 are genes that indicate the production of collagen type 1 and collagen type 3, respectively, while TGF-β1 is a gene that represents the level of inflammatory response. The results showed that the production of collagen type 1 was significantly higher in the PDO thread group than in the HA thread group. On the other hand, the expression level of collagen type 3 was higher in the HA thread group, but the difference was not statistically significant. The PDO thread group showed a statistically significant increase in TGF-β1 compared to the HA thread group, indicating a higher level of inflammatory response (Fig. 6).

## Discussion

The use of threads has made significant advancements and is being applied with various materials. Prominent materials used in thread-based procedures include PDO, PLA, and PLLA, among others, in their filament form [7]. The HA used in the form of threads is typically coated with an absorbable material, and the percentage of HA varies depending on the product type [12]. To date, there have been no products available that utilize 100% pure HA in a solid form for injectable purposes. Regarding the physical properties of HA fillers, a multitude of products have been developed in conjunction with extensive research studies [13]. Differences in manufacturing processes, encompassing factors such as the extent of crosslinking, conditions of crosslinking, initial molecular weight of the HA, and subsequent post-crosslinking modifications possess the potential to influence filler characteristics [14–16]. Despite these numerous studies, there has been a lack of in vivo studies conducted on solid-type HA. This study showed the safety and efficacy of solid-type HA thread in vivo experiment, which were compared to conventional gel-type HA filler and PDO thread.

PDO, PLA, and PLLA are inserted in thread form with the purpose of promoting collagen synthesis. PDO in the subcutaneous tissue acts as collagen stimulant, one study showed that type 1 collagen formation increased for 7 months [17, 18]. Recent study of Shin et al. reported that mono-PLA threads showed better results than mono-PDO thread [9]. While these kinds of threads have been proven to be safe in various studies, there are rare instances where they can lead to complications such as cellulitis and abscess formation [19]. These complications, as demonstrated in our experiment, can be attributed to inflammatory reactions occurring around PDO threads. Cabral et al. reported that the use of dermal PLLA filler was found to have a potentially adverse impact on the fibroblast phenotype, which may lead to clinical complications [20]. In contrast, HA threads did not elicit such reactions, confirming their safety.

The conventional gel-type HA filler frequently encountered difficulties in achieving precise injections at the desired locations due to variations in the characteristics of the surrounding tissue [21, 22]. Furthermore, manual molding following the injection was frequently required, and the molding process posed challenges in certain cases [23]. In the present study, the investigation of solid-type HA presents a promising alternative for addressing these challenges. HA threads are precisely inserted into targeted areas, eliminating concerns about unexpected material migration. Unlike HA fillers, HA threads work differently by attracting and retaining water, resulting in a volumizing effect. While further research is required to determine the extent of volume increase, the use of solid HA threads holds promise for more predictable outcomes. Although rare, ischemic complications have been reported in cases of conventional gel-type HA injections [24]. Injection necrosis can occur due to the interruption of vascular supply, which can be caused by either compression or complete blockage of blood vessels resulting from the direct injection of the material into the vessel. Solid-type thread HA offers the advantage of being insertable without the associated concerns of vascular complications.

There may be concerns regarding the use of solid-type HA due to its higher density compared to gel-type fillers. Recent studies have reported that even with the same HA filler, variations in inflammatory response can arise depending on crosslinking and application methods [15, 25]. However, in vivo experiments have demonstrated its safety profile without causing inflammatory changes. In clinical settings, solid-type HA fillers are anticipated to yield skin rejuvenation effects rather than immediate volume augmentation observed with gel-type fillers [26]. Nevertheless, it is difficult to directly extrapolate the results of animal experiments to human applications. Thus, additional clinical research is warranted to evaluate and confirm these effects.

This study has several limitations. First, the findings based on animal experiments alone may not be sufficient to determine the complete clinical application guidelines for solid-type HA fillers, considering their different physical properties. Using a different species for more efficient observations, yet employing the same species and the same number of animals, would have allowed for a more parallel comparison. In addition, differences in collagen synthesis will require statistical verification through experiments involving an adequate number of animals. While our current follow-up period provided valuable insights into the short-term effects and immediate outcomes of the procedures, a longer follow-up would be necessary to gather more comprehensive data on sustainability and to monitor for any delayed adverse reactions. Further research is needed in a clinical setting to establish the specific usage guidelines for these products in real-world applications.

## Conclusion

In conclusion, this in vivo study demonstrated the stable application of solid-type pure HA threads and their potential for inducing collagen production compared to HA filler, while also yielding a low inflammatory response compared to PDO threads. The results of this study provide valuable insights into the potential applications of solid-type pure HA in the field of cosmetic dermatology.
