# Correction: Comparative In Vivo Study of Solid-Type Pure Hyaluronic Acid in Thread Form: Safety and Efficacy Compared to Hyaluronic Acid Filler and Polydioxanone Threads

**DOI:** 10.1007/s00266-023-03710-7

**Published:** 2023-10-23

**Authors:** Jong-Ho Kim, Man Wong Han, Myoung-Han Lee, Dong-Keon Kweon, Young Jin Park, Chan Yeong Heo

**Affiliations:** 1 Department of Plastic and Reconstructive Surgery, Seoul National University College of Medicine, Seoul National University Bundang Hospital, 82 Gumi-ro 173beon-gil, Bundang-gu, Seongnam, 463-707 Korea; 2 Jinwoo Bio Co. Ltd., Yongin si, Korea; 3 Ksamsung Plastic Surgery, Seoul, Korea

**Correction to: Aesth Plast Surg** 10.1007/s00266-023-03614-6

The article “Comparative In Vivo Study of Solid-Type Pure Hyaluronic Acid in Thread Form: Safety and Efficacy Compared to Hyaluronic Acid Filler and Polydioxanone Threads”, written by Jong-Ho Kim, Man Wong Han, Myoung-Han Lee, Dong-Keon Kweon, Young Jin Park, and Chan Yeong Heo, was originally published electronically on the publisher’s internet portal on 29 August 2023 without open access. With the author(s)’ decision to opt for Open Choice the copyright of the article changed on 20 September 2023 to © The Authors 2023 and the article is forthwith distributed under a Creative Commons licence CC BY. This licence allows readers to copy, distribute and transmit the Article as long as it is attributed back to the author. Readers are permitted to alter, transform or build upon the Article for commercial purposes http://creativecommons.org/licenses/by/4.0. This research was funded by the Ministry of Trade, Industry, and Energy (MOTIE), Korea, under the “Industrial Innovation Infrastructure Establishment Project”(reference number P0018140) supervised by the Korea Institute for Advancement of Technology (KIAT) and was supported by a grant of the 2023 Commercialization Joint Research Association Operation Support Project through the Korea Health Industry Development Institute (KHIDI), funded by the Ministry of Health & Welfare, Republic of Korea.

